# Employer-Sponsored Health Insurance for Workers in the Hourly Service Sector

**DOI:** 10.1001/jamahealthforum.2025.4747

**Published:** 2025-11-07

**Authors:** Gabriella Aboulafia, Daniel Schneider

**Affiliations:** 1 Harvard University PhD Program in Health Policy, Cambridge, Massachusetts; 2 Harvard Kennedy School of Government, Faculty of Arts and Sciences, Harvard University, Cambridge, Massachusetts

## Abstract

This survey study uses employer-employee linked survey data to assess the mechanisms associated with exclusion of workers in the hourly service sector from health insurance coverage.

## Introduction

Current policy debate often assumes that large employers offer health insurance to their workers. In defending Medicaid cuts in HR 1, policymakers have argued that Medicaid coverage losses would be offset by gains in employer-sponsored insurance (ESI).^1^ This may seem plausible: the Affordable Care Act (ACA) employer shared responsibility provision requires that employers offer affordable minimum-value coverage or potentially face penalties.^2^ But exceptions to this provision limit its reach, especially in the service sector, where many workers with low-wage jobs are concentrated.

First, while service-sector firms might be assumed to have shared responsibility, such firms and many others employ workers through independent franchises that do not meet the ACA 50-worker cutoff that exempts employers from responsibility. Second, employees working fewer than 30 hours a week are also exempt. In the service sector, part-time status is widespread and often involuntary.^3^ Third, employers may impose a 90-day waiting period and a 12-month lookback period to assess compliance with the hours threshold.^4^ In a labor market characterized by high turnover, workers may not stay long enough to qualify.^5^ We use novel employer-employee linked survey data to show how these mechanisms may be associated with exclusion from coverage.

## Methods

We used data from the Shift Project, a biannual survey of workers 18 and older employed at more than 150 of the largest US retail and food service firms. Respondents reported detailed information on hours worked, tenure, firm ownership, and access to ESI. A discussion of The Shift Project data collection, methodology, and data validation is available in Schneider and Harknett^6^ and the eMethods in Supplement 1. Our analysis included pooled data from hourly workers surveyed in 2023 to 2024 (eMethods in Supplement 1). We applied weights to adjust the demographic characteristics of the sample to match the survey-weighted demographic characteristics of service-sector workers in the Census Bureau American Community Survey (eMethods in Supplement 1). The study followed AAPOR reporting guideline and was approved by the Harvard University Committee on the Use of Human Subjects. Participants provided informed consent.

We first examine the share of workers in exemption categories under the employer provision: part-time status, short tenure, and employment at franchised establishments, used as a proxy for firm size (eTable in Supplement 1). The Shift Project validates firm ownership status using a state-level firm franchise status measure based on administrative data (eMethods in Supplement 1). We then estimate a linear probability model to assess the association between each exemption category and being offered ESI, adjusting for demographic characteristics and state and year fixed effects (eMethods in Supplement 1) and compute predicted probabilities for ESI access. Data were analyzed using Stata version 18.0 (StataCorp) and statistical significance was assessed at 2-sided *P* < .05.

## Results

Of pooled data from 19 885 hourly workers (weighted 52.4% female; weighted 46.2% aged 18-29 years), large shares of workers in the hourly service sector occupy the employer provision exemption categories (Figure). Nearly 30% were part-time, 26% worked at a franchised firm, and 17% had short tenure. More than one-half (54%) were excluded as members of at least one category.

**Figure.**
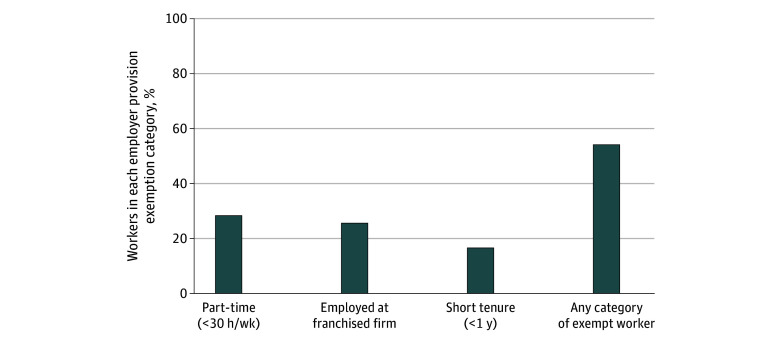
Workers in the Hourly Service Sector by Employer Provision Exemption Category Figure shows share of hourly service-sector workers within each employer provision exemption category. Data are pooled survey data from The Shift Project, 2023-2024 (n = 19 885). Survey weights to adjust the demographic characteristics of the Shift sample to match the survey-weighted demographic characteristics of service sector workers in the Census Bureau American Community survey were applied.

Workers in the exemption categories are far less likely to be offered ESI than those protected by the provision (Table). Workers in the hourly service-sector are 29 percentage points (95% CI, −31 to −26 percentage points) less likely to be offered ESI if they are at a franchised firm, 25 percentage points (95% CI, −28 to −23 percentage points) less likely if part-time, and 8 percentage points (95% CI, −11 to −5.8 percentage points) less likely if short-tenured.

**Table.** Association Between Employer Provision Exemption Categories and Employer-Sponsored Insurance Offerings^a^

Exemption category	Unweighted						Weighted					
	Unadjusted			Adjusted			Unadjusted			Adjusted		
	Probability offered ESI, %	Difference (95% CI)	*P* value	Predicted probability offered ESI, %	Difference (95% CI)	*P* value	Probability offered ESI, %	Difference (95% CI)	*P* value	Predicted probability offered ESI, %	Difference (95% CI)	*P* value
Franchise status												
Franchised	44.4	−0.29 (−0.31 to −0.28)	<.001	45.7	−0.28 (−0.29 to −0.27)	<.001	44.7	−0.30 (−0.33 to −0.28)	<.001	46.1	−0.29 (−0.31 to −0.26)	<.001
Corporate-owned	74.3	[Reference]	NA	73.8	[Reference]	NA	75.1	[Reference]	NA	74.7	[Reference]	NA
Part-time status												
<30 h/wk	45.2	−0.29 (−0.31 to −0.28)	<.001	45.4	−0.29 (−0.30 to −0.27)	<.001	48.3	−0.27 (−0.29 to −0.24)	<.001	49.1	−0.25 (−0.28 to −0.23)	<.001
At least 30 h/wk	74.3	[Reference]	NA	74.2	[Reference]	NA	74.9	[Reference]	NA	74.6	[Reference]	NA
Tenure												
<1 y	55.1	−0.13 (−0.15 to −0.12)	<.001	60.1	−0.07 (−0.09 to −0.06)	<.001	55.6	−0.14 (−0.17 to −0.11)	<.001	60.3	−0.08 (−0.11 to −0.06)	<.001
At least 1 y	68.4	[Reference]	NA	67.3	[Reference]	NA	69.7	[Reference]	NA	68.8	[Reference]	NA

Abbreviations: ESI, employer-sponsored insurance; NA, not applicable.

^a^ Table reports results from linear probability models examining factors associated with access to employer-sponsored insurance; predicted probabilities were estimated using the Stata margins postestimation command. Unadjusted results are from bivariate models assessing the association between each exemption category and ESI access; adjusted results are from a multivariate model including all controls and fixed effects. The full list of controls and fixed effects is available in eMethods in Supplement 1. Data are pooled survey data from the Shift Project, 2023-2024 (n = 19 885). Unweighted results and results weighted to adjust the demographic characteristics of the Shift sample to match the survey-weighted demographic characteristics of service-sector workers in the Census Bureau American Community Survey are reported.

## Discussion

The structure of the low-wage labor market coupled with the limitations of the employer provision leaves many workers in the service sector without access to ESI. A limitation of this study is that it does not examine the affordability of ESI offers. Some ESI plans may meet the ACA’s definition of affordable coverage but remain effectively unaffordable to workers. These realities have important implications for policies that view ESI as a substitute for Medicaid for workers in the service sector.

## Notes

Supplement 1.
eMethods
**eTable.** Main variable question wording and coding
eReferences

Supplement 2.
Data Sharing Statement
