# Supplementary material for: Employer-Sponsored Health Insurance for Workers in the Hourly Service Sector
Source: JAMA Health Forum. 2025 Nov 7;6(11):e254747. doi: 10.1001/jamahealthforum.2025.4747 (PMC12595526; doi:10.1001/jamahealthforum.2025.4747)
Supplement: Supplement 1. — eMethods eTable. Main variable question wording and coding eReferences [file jamahealthforum-e254747-s001.pdf]

Supplemental Online Content

Aboulafia G, Schneider D. Employer-sponsored health insurance for workers in the hourly service sector. *JAMA Health Forum.* 2025;6(11):e254747. doi:10.1001/jamahealthforum.2025.4747

eMethods

eTable. Main variable question wording and coding

eReferences

This supplemental material has been provided by the authors to give readers additional information about their work.

© 2025 Aboulafia G et al. *JAMA Health Forum*.

eMethods.

a) Sample details

This paper draws on survey data collected by The Shift Project between March 2023 and November 2024 from hourly service sector workers employed at one of 168 of the largest retail and food service employers. The survey data collection was national in scope and the survey sample includes respondents from all 50 U.S. states and Washington, D.C. The Shift Project data are collected using a novel online sampling and recruitment approach. To reach a target population of hourly workers employed at large firms in the service sector, Shift first identified a set of firms by drawing from the National Retail Federation's list of top 100 retailers[1] and the Restaurant Business' list of top 100 restaurant chains. Shift then turned to Meta's targeted advertising platform, which permits advertisers to construct "audiences" of Facebook and Instagram users with specific attributes, including employer. Shift cross-referenced the lists of large service-sector firms with the employers available on Meta's platform and constructed employer-specific audiences of users employed at those firms. While not all companies can be targeted on Meta, Shift successfully sampled 44 of the top 50 retailers and 48 of the top 50 restaurants. The Shift data also sample respondents at 135 other large companies that are a purposive mix of retailers in the 50-100 rank, restaurants in the 50-100 rank, as well as large companies in hospitality and logistics that are not represented on the National Retail Federation or Restaurant Business lists. A subset of these companies is surveyed at every wave, with other companies rotating in and out of sample purposively depending on the wave.

At each wave of data collection, Shift delivered paid survey recruitment advertisements to Facebook and Instagram users in these audiences inviting them to take an online survey. The advertisements are employer-specific, using variants on the message, "Survey for [EMPLOYER NAME] workers" and the advertisements, consent, and survey branding make clear that the surveys are University-sponsored research. The Shift cross-sections are fielded twice annually, once in the Spring between March and June and once in the Fall between September and November. At each wave, a new sample of workers is recruited using the method. This approach has the advantage of producing a broad cross-section of American service workers who answer detailed questions about their working conditions and wellbeing, at a feasible cost.

However, the data are not constructed using a probability sampling method. In methodological work, Schneider and Harknett (2022) show that Shift reproduces both univariate statistics and associations that mirror those found for similar workers in gold-standard probability data sets[2] and other work shows that Shift Project estimates can effectively capture announced company changes in practice and responses to public policy mandates.[3–6]

While the Shift Project includes 17 repeated cross-sections collected between January of 2016 and November of 2024, not all measures are available at all waves. Our key dependent variable, employer offer of health insurance, was collected beginning at

© 2025 Aboulafia G et al. *JAMA Health Forum*.

wave 3 and our direct-report of franchise ownership was collected beginning at wave 14. In our analysis, we then draw on data collected between March of 2023 (the start of wave 14) and November of 2024 (the end of wave 17), the most recent wave of data. We limit our main analysis sample to the 19,885 respondents who were paid hourly and who had non-missing data on all of our model covariates (noted below).

The Shift Project data uniquely contain reports of ESI offer, job tenure and usual work hours, and information on franchising for a large sample of the policy-relevant population of low-wage workers in the service-sector.

b) Weighting to the American Community Survey

We construct survey weights to adjust the demographic characteristics of the Shift survey sample to match the survey-weighted demographic characteristics of service-sector workers in the American Community Survey (ACS) for the years 2021-2023. We align the ACS sample with the Shift sample by selecting workers in the ACS who are employed in the same occupations and industries as the Shift sample. These weights are constructed using age, gender, race/ethnicity, educational attainment, and state of residence, and are applied throughout our analysis.

To assess the sensitivity of our estimates to not weighting the data, we run our main model **(manuscript Table)** with and without weights, and find the estimates are very stable.

c) Main model specification

To measure the association between being offered employer-sponsored insurance (ESI) and falling into an employer shared responsibility provision exemption category, we draw on the following linear probability model that regresses a dichotomous measure of being offered ESI on franchise ownership, part-time status, short tenure, and individual-level covariates and state and year fixed effects (i indexes individuals, s states, and t years).

$$benefits\_health_{ist} = \alpha + \beta_1 franchise_{ist} + \beta_2 lessthan30_{ist} + \beta_3 lessthanyr_{ist} + \beta_k \boldsymbol{X}_{ist} + \gamma_s + \delta_t + \epsilon_{ist}$$

In our model, $benefits\_health$ is a dichotomous measure for whether the respondent is offered ESI. $franchise$, $lessthan30$, and $lessthanyr$ are binary indicators for whether an individual works at franchised firm, works less than 30 hours/week, and has been at their current employer for less than a year, respectively. (Question wording and coding for our primary outcome and explanatory variables are included below in **eTable 2**). $\boldsymbol{X}$ is a vector of individual-level covariates including: self-reported race/ethnicity (Black, non-Hispanic; Hispanic; other race/ethnicity [which includes those who identify as American Indian or Alaskan Native, Asian or Pacific Islander, those who selected "other," or those who selected two or more race and/or ethnicities]; and white); sex

© 2025 Aboulafia G et al. *JAMA Health Forum*.

(male; female); age; whether the respondent has a child; whether the respondent is married, cohabiting, or not living with a partner; and educational attainment (no high-school degree; high-school degree or GED; or some college and above). We also include state (γ_s) and year (δ_t) fixed effects.

d) Validating self-reported firm ownership status

Our analysis uses direct worker reports of whether the establishment at which they work is owned by a franchisee. We validate this approach by drawing on administrative data on franchising. Franchising firms must file Franchise Disclosure Documents (FDD) with the Federal Trade Commission each year. These filings include tables that report on the number of franchised establishments and company-owned establishments in each state. We obtained by purchase and by FOIA request to state authorities PDF files of the FDDs filed by the franchising firms represented in the Shift Project sample. We then scraped these files to produce numeric data on the counts of franchised and company-owned establishments which we use to calculate the percent of establishments that are franchised at each firm.

We then assess the firm-level correspondence between the measure of franchising derived above from the FDDs with the direct-report measure used in our primary model. The pair-wise correlation is 0.97, suggesting the FDD and direct-report measures are extremely consistent with each other.

© 2025 Aboulafia G et al. *JAMA Health Forum*.

eTable. Main variable question wording and coding

Variables	Question wording	Coding
**Health insurance offered**	Please look at the following list of benefits that employers sometimes make available to their employees.<br>Which of the benefits on this list can you receive as part of your job at [EMPLOYER NAME]? Please mark all that apply.<br>1 Paid sick days<br>2 Paid vacation days<br>3 Health plan or medical insurance<br>4 Dental benefits<br>5 Paid maternity or paternity leave<br>6 Unpaid maternity or paternity leave which would allow you to return to the same job, or one similar to it<br>7 A retirement plan other than Social Security<br>8 Tuition reimbursement for certain types of schooling<br>9 Company-provided or subsidized child-care<br>10 None of these	"3" = 1;<br>Otherwise, 0
**Part-time status**	How many hours per week do you usually work at [EMPLOYER NAME]? Please enter a number between 0 and 80 hours per week.<br>_______	< 30 hours = 1;<br>>= 30 hours = 0
**Short tenure**	How long have you been working at [EMPLOYER NAME]?<br>1 Less than 1 year<br>2 1 year<br>3 2 years<br>4 3 years<br>5 4 years<br>6 5 years<br>7 6 years<br>8 7 years<br>9 8 years<br>10 9 years<br>11 10 or more years<br>12 Don't know/refuse	"1" = 1;<br>"2"-"11" = 0;<br>"12" = missing
**Employed at franchise**	Who is the owner of the [EMPLOYER NAME] where you work?<br>1 My workplace is owned by [EMPLOYER NAME] directly<br>2 My workplace is owned by a franchisee<br>3 Don't know/refuse	"1" = 0;<br>"2" = 1;<br>"3" = missing

© 2025 Aboulafia G et al. *JAMA Health Forum*.

eReferences.

1. Top 100 Retailers 2024 List. National Retail Federation; 2024 [cited 2025 June 18]. Available from: https://nrf.com/research-insights/top-retailers/top-100-retailers/top-100-retailers-2024-list

2. Schneider D, Harknett K. What's to Like? Facebook as a Tool for Survey Data Collection. Sociological Methods & Research. 2022 Feb 1;51(1):108–40.

3. Schneider D. Paid Sick Leave in Washington State: Evidence on Employee Outcomes, 2016–2018. Am J Public Health. 2020 Apr;110(4):499–504.

4. Schneider D, Harknett K, Vivas-Portillo E. Olive Garden's Expansion Of Paid Sick Leave During COVID-19 Reduced The Share Of Employees Working While Sick. Health Affairs. 2021 Aug;40(8):1328–36.

5. Harknett K, Schneider D, Irwin V. Improving health and economic security by reducing work schedule uncertainty. Proceedings of the National Academy of Sciences. 2021 Oct 19;118(42):e2107828118.

6. Harknett K, Schneider D. Mandates Narrow Gender Gaps In Paid Sick Leave Coverage For Low-Wage Workers In The US. Health Affairs. 2022 Nov;41(11):1575–82.

© 2025 Aboulafia G et al. *JAMA Health Forum*.
