# Supplementary material for: Employer-Sponsored Health Insurance for Workers in the Hourly Service Sector
Source: JAMA Health Forum. 2025 Nov 7;6(11):e254747. doi: 10.1001/jamahealthforum.2025.4747 (PMC12595526; doi:10.1001/jamahealthforum.2025.4747)
Supplement: Supplement 2. — Data Sharing Statement [file jamahealthforum-e254747-s002.pdf]

Data Sharing Statement

Aboulafia. Employer-Sponsored Health Insurance for Workers in the Hourly Service Sector. *JAMA Health Forum*. Published November 07, 2025. doi:10.1001/jamahealthforum.2025.4747

Data
Data available: No
